# Printing Structurally Anisotropic Biocompatible Fibrillar Hydrogel for Guided Cell Alignment

**DOI:** 10.3390/gels8110685

**Published:** 2022-10-22

**Authors:** Zhengkun Chen, Nancy Khuu, Fei Xu, Sina Kheiri, Ilya Yakavets, Faeze Rakhshani, Sofia Morozova, Eugenia Kumacheva

**Affiliations:** 1 Department of Chemistry, University of Toronto, Toronto, ON M5S 3H6, Canada; 2 Department of Mechanical and Industrial Engineering, University of Toronto, Toronto, ON M5S 3G8, Canada; 3 N.E. Bauman Moscow State Technical University, 5/1 2nd Baumanskaya Street, 105005 Moscow, Russia; 4 Department of Chemical Engineering and Applied Chemistry, University of Toronto, Toronto, ON M5S 3E5, Canada; 5 The Institute of Biomedical Engineering, University of Toronto, Toronto, ON M5S 3G9, Canada

**Keywords:** anisotropy, cellulose nanofiber, 3D printing, hydrogels, alignment

## Abstract

Many fibrous biological tissues exhibit structural anisotropy due to the alignment of fibers in the extracellular matrix. To study the impact of such anisotropy on cell proliferation, orientation, and mobility, it is important to recapitulate and achieve control over the structure of man-made hydrogel scaffolds for cell culture. Here, we report a chemically crosslinked fibrous hydrogel due to the reaction between aldehyde-modified cellulose nanofibers and gelatin. We explored two ways to induce structural anisotropy in this gel by extruding the hydrogel precursor through two different printheads. The cellulose nanofibers in the hydrogel ink underwent shear-induced alignment during extrusion and retained it in the chemically crosslinked hydrogel. The degree of anisotropy was controlled by the ink composition and extrusion flow rate. The structural anisotropy of the hydrogel extruded through a nozzle affected the orientation of human dermal fibroblasts that were either seeded on the hydrogel surface or encapsulated in the extruded hydrogel. The reported straightforward approach to constructing fibrillar hydrogel scaffolds with structural anisotropy can be used in studies of the biological impact of tissue anisotropy.

## 1. Introduction

Many biological tissues exhibit structural anisotropy because the constituents of the extracellular matrix (ECM) and/or cells are oriented along a particular axis [1]. Structural anisotropy imparts tissues with orientation-specific properties, e.g., directional forces [2,3], response to deformation [4], or unidirectional electrical signal transmission [5]. Structural anisotropy also plays an important role in the wound-healing process by promoting fibroblast transformation into a myofibroblast phenotype [6], which accelerates wound closure [7].

In many anisotropic tissues, such as cornea, cartilage, bone, and skin, structural anisotropy originates from the directional orientation of ECM fibers [8,9,10,11,12]. For example, in the dermis layer of the skin, the directional orientation of collagen fibers leads to anisotropy in the mechanical properties of the skin [13]. In tendons, collagen fibers are oriented along the long axis of the tendon to withstand load and transmit force from skeletal muscles to bone [14]. In bones, collagen fibers are organized into lamellae that are further organized into a rotated plywood structure, which imparts bone with mechanical anisotropic properties [15]. The important role of structural anisotropy in regulating tissue mechanical properties and cell behavior stimulates the development of structurally anisotropic tissue scaffolds, which not only provide a biomimetic tissue scaffold for cell culture applications but also act as a platform to investigate the role of anisotropy on cell behavior.

Hydrogels have promising applications as scaffolds for cell culture, owing to the high water content and the capability of chemical and/or physical modification to recapitulate the biochemical and biophysical properties of native ECM [16]. Cellulose nanofibers (CNFs) are a cost-effective material for fabricating hydrogels [17,18,19]. The biocompatibility of CNFs has been demonstrated by their applications for cell culture [20,21,22,23]. The bundling of CNFs in hydrogels offers a simple approach to creating a biomimetic fibrillar structure that is characteristic of many living tissues [24]. Compared to fibrillar hydrogels created by electrospinning, the formation of fibrillar hydrogels via hierarchical assembly of CNFs circumvents the use of toxic solvents and complex instrumentation [25]. Furthermore, the ability to control the orientation of CNF fibers in hydrogels enables the induction of structural anisotropy [26]. Structural anisotropy can be induced in CNF-based hydrogels by directional freeze-casting [27], stimulus of magnetic or electric fields [28], or electrospinning [29]. However, freeze-casting and electrospinning involve harsh conditions during the fabrication and cannot be used for in situ cell encapsulation in 3D cell culture. Similarly, long-term exposure of cells to magnetic or electric fields may affect cell viability and proliferation. In addition, the aforementioned methods require the use of a specific instrument and lack versatility to fabricate complex 3D structures [30].

Extrusion-based 3D printing is a straightforward method that can be used for the shear-induced alignment of CNFs in hydrogels. It does not require a sophisticated experimental setup and enables construction of 3D architectures. Previously, CNF-based materials have been used in extrusion-based 3D printing to enhance the mechanical properties of “inks” [18,31] and for printing hydrogel actuators [32] and sensors [33]. Yet, constructing structurally anisotropic CNF hydrogels for cell culture via extrusion-based 3D printing has not been reported. Given the accumulating interest in exploring the biological impact of structural anisotropy, a simple and effective approach to the fabrication of structurally anisotropic hydrogels suitable for both 2D cell culture and 3D cell encapsulation is in great demand.

In the present work, we report the use of a two-component ink derived from an aqueous suspension of aldehyde-modified CNFs (a-CNFs) and gelatin to fabricate structurally anisotropic hydrogels. The a-CNFs served as the building block to generate a fibrillar hydrogel, while gelatin provides the cell adhesion ligands. The ink was extruded through a microfluidic multichannel printhead or a nozzle. The extent of structural anisotropy in the hydrogels was varied by changing the flow rate of the ink during extrusion and the concentration of a-CNFs in the ink. To explore the influence of the anisotropic structure on cell alignment, we studied the orientation of human dermal fibroblasts (HDFs) spreading either on the extruded hydrogel or within the hydrogel. By comparing the degree of cell alignment on or in the hydrogels fabricated via the two different approaches, we showed that the hydrogels extruded through a nozzle were more effective in governing cell alignment.

## 2. Results and Discussion

### 2.1. Preparation of a-CNF/Gelatin Ink

**Figure 1:**
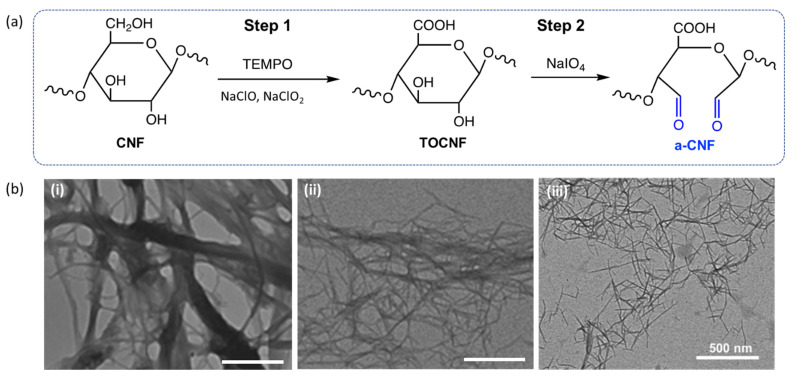
Surface modification of CNFs. (**a**) Schematic of two-step modification of CNFs with carboxyl groups (Step 1) and aldehyde groups (Step 2). (**b**) TEM images of pristine CNFs (i), TOCNFs (ii) and a-CNFs (iii). Scale bars are 500 nm.

Hydrogen bonding between pristine CNFs yielded thick bundles of aggregated CNFs with poor dispersibility in water. To overcome this problem, the primary hydroxyl groups on the surface of CNFs were oxidized to carboxyl groups using 2,2,6,6-tetramethyl-1-piperidinyloxy (TEMPO) modification, as described elsewhere [34]. The remaining hydroxyl groups on the TEMPO-modified CNFs (TOCNF) were then reacted with sodium periodate to introduce aldehyde groups to the TOCNF surface [35]. Figure 1a shows the reaction scheme of the surface modification of CNFs with TEMPO, leading to the formation of TOCNFs, followed by the modification of TOCNFs to introduce aldehyde groups on their surface. Later in the text, we refer to these aldehyde-functionalized TOCNFs as a-CNFs.

The dissociation of CNF aggregates into individual fibers after their treatment with TEMPO was confirmed by ultraviolet-visible (UV-vis) spectroscopy (Figure S1a, Supporting Information) and transmission electron microscopy (TEM) imaging. Following TEMPO modification, the transmittance of the 0.1 wt.% suspension of pristine CNFs increased from 4.3 to 78% at 400 nm and from 11.4 to 87.8% at 600 nm due to the dissociation of CNF bundles. Figure 1b shows representative TEM images of pristine CNFs, TOCNFs, and a-CNFs. While pristine CNFs formed thick bundles, TOCNFs formed fibers with an average diameter of 13.8 ± 2.4 nm (Figure S1b, Supporting Information), consistent with the reported diameter of individual CNFs [36,37].

Further modification of TOCNFs with sodium periodate was confirmed in attenuated total reflectance–Fourier transform infrared (ATR-FTIR) experiments. The appearance of a peak at 1700 cm^−1^ in the spectrum of the TOCNFs corresponded to the carbonyl stretch of the aldehyde groups and confirmed the functionalization of the TOCNF surface (Figure S2, Supporting Information). The molar concentration of surface aldehyde groups on a-CNFs was determined by their titration with hydroxylamine hydrochloride (Table S1, Supporting Information). To limit the quantity of NaIO_4_ used while maximizing the number of aldehyde groups on the a-CNFs, a 1.0:1.0 TOCNF: NaIO_4_ mass ratio and 24 h reaction time were used in all subsequent experiments. Under these conditions, the molar concentration of aldehyde groups on the a-CNFs was 2.6 mmol/g. The TEM image of a-CNFs showed that following TOCNF modification with aldehyde groups, the a-CNFs existed as individual fibers (Figure 1b(iii)).

### 2.2. Properties of a-CNF/Gelatin Hydrogels

An ink for extrusion-based 3D printing was prepared by mixing an aqueous suspension of a-CNFs with an aqueous solution of gelatin to reach the concentration of a-CNF, C_a-CNF_, of 0.25, 0.33, 0.50, or 1.0 wt.% at the concentration for gelatin, C_gelatin_, of 4 wt.%. As the concentration of aldehyde groups on a-CNFs was 2.6 mmol/g and the concentration of amine groups of gelatin was 0.213 mmol/g [35], the ratios of mass concentrations of a-CNFs and gelatin corresponded to the ratio of molar concentrations of aldehyde groups to amine groups of 0.75:1.0, 1.0:1.0, 1.5:1.0, and 3.0:1.0, respectively.

**Figure 2:**
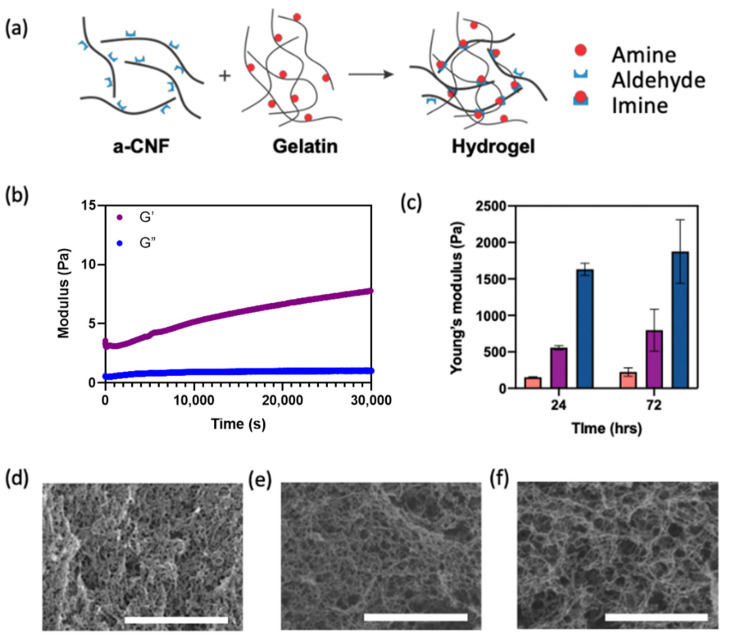
Properties of a-CNF/gelatin hydrogels. (**a**) Schematic of gel formation from a-CNFs and gelatin. (**b**) Rheological characterization of gel formation from the a-CNF/gelatin mixture at 37 °C, depicting the variation in storage modulus (red) and loss modulus (blue). C_a-CNF_ = 0.50 wt.%; C_gelatin_ = 4.0 wt.%. (**c**) Variation in the compression Young’s modulus of a-CNF/gelatin hydrogels at 37 °C at C_a-CNF_ in the hydrogels of 0.33 wt.% (orange), 0.50 wt.% (purple), and 1.0 wt.% (blue). C_gelatin_ = 4.0 wt.%. (**d**–**f**) Scanning electron microscopy (SEM) images of a-CNF/gelatin hydrogels at C_a-CNF_ of 0.33 wt.% (**d**), 0.50 wt.% (**e**) and 1.0 wt.% (**f**) at C_gelatin_ = 4.0 wt.%. Scale bars are 2 μm.

Figure 2a depicts the preparation of covalently crosslinked a-CNF/gelatin hydrogels. The Schiff-base reaction between the aldehyde groups on the surface of a-CNFs and the primary amine groups of gelatin resulted in the formation of the covalently crosslinked gel. In addition, the entanglement of a-CNFs, hydrogen bonding between hydroxyl groups on a-CNFs and amine groups of gelatin, and electrostatic interactions between negatively charged carboxyl groups of a-CNFs and positively charged amine groups of gelatin led to the formation of a physically crosslinked gel.

Gelation of the a-CNF/gelatin mixed suspension with varying C_a-CNF_ was confirmed in rheology experiments conducted at 37 °C. The variation in the storage modulus, G′, and loss modulus, G″, was examined in time sweep experiments for a-CNF/gelatin mixtures with C_a-CNF_ of 0.25, 0.33, 0.50, or 1.0 wt.% at C_gelatin_ = 4.0 wt.%. Gelation in rheology experiments was confirmed when G′ exceeded G″ [38].

When the molar ratio of aldehyde groups to amine groups was 0.75:1.0, gelation was not observed for 8 h, owing to a limited number of aldehyde groups on the a-CNF surface and thus, an insufficient density of Schiff-base crosslinks (Figure S3a, Supporting Information). At the molar ratio of aldehyde groups to amine groups of 1.0:1.0, 1.5:1.0, or 3.0:1.0, gelation occurred after 5200 s at C_a-CNF_ = 0.33 wt.% (Figure S3b, Supporting Information) and immediately after mixing a-CNFs and gelatin at C_a-CNF_ of 0.50 or 1.0 wt.% (Figure 2b and Figure S3c, Supporting Information).

Figure 2c shows the variation in the compression Young’s modulus of hydrogels with varying C_a-CNF,_ measured 24 and 72 h after mixing a-CNFs and gelatin. The Young’s modulus measured 24 h after mixing increased from 150.0 ± 7.6 to 1632.0 ± 83.6 Pa, when C_a-CNF_ increased from 0.33 to 1.0 wt.%. As the corresponding ratios of molar concentrations of aldehyde groups on a-CNFs to amine groups of gelatin were 1.0:1.0, 1.5:1.0 or 3.0:1.0, the crosslinking density was not expected to change, since the molar concentration of amine groups remained constant. In contrast, the increase in Young’s modulus was observed at higher C_a-CNF,_ due to stronger a-CNF entanglement and intermolecular interactions between the a-CNF fibers. After 72 h, the Young’s modulus increased from 223.0 ± 57.0 Pa to 1875.0 ± 435 Pa, with C_a-CNF_ increasing from 0.33 to 1.0 wt.%, indicating that no significant change in mechanical properties occurred over the subsequent 2 days.

The structure of cast hydrogel with C_a-CNF_ of 0.33, 0.50 and 1.0 wt.%, respectively, was visualized using SEM (Figure 2d–f). At a higher C_a-CNF_, the hydrogel exhibited a more pronounced filamentous structure and larger pore size.

### 2.3. Structural Anisotropy of Extruded Hydrogel Sheets

**Figure 3:**
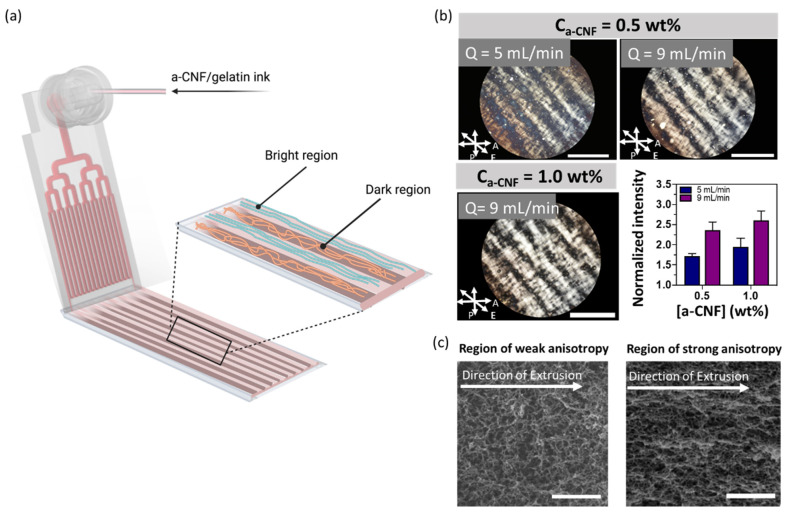
Microfluidic extrusion-based printing of structurally anisotropic hydrogel sheets. (**a**) Schematic depicting printing of a hydrogel sheet. (**b**) POM images of the extruded hydrogel at C_gelatin_ = 4.0 wt.%. Top left: C_a-CNF_ = 0.50 wt.%, Q = 5.0 mL/min. Top right: C_a-CNF_ = 0.50 wt.%, Q = 9.0 mL/min. Bottom left: C_a-CNF_ = 1.0 wt.%, Q = 9.0 mL/min. Scale bars are 2 mm. Bottom right: Average light intensity of the bright stripes normalized to the dark stripes at different C_a-CNF_ (C_gelatin_ = 4.0 wt.%) and flow rates Q. Each bar represents three independently examined hydrogels. The arrows in the image depict the direction of extrusion (E) and the direction of the polarizer (P) and analyzer (A) with respect to the direction of extrusion. (**c**) SEM images of extruded hydrogel sheets in the region of weak and strong anisotropy. The gel was extruded at Q = 9.0 mL/min. C_a-CNF_ = 1.0 wt.%; C_gelatin_ = 4.0 wt.%. Scale bars are 1 μm.

In the first approach, we extruded a-CNF/gelatin hydrogel sheets using a microfluidic printhead mounted on a 3D printer [39,40,41,42]. The printhead consisted of 4 generations of bifurcated parallel microchannels, with 16 channels reaching the outlet of the printhead (Figure S4, Supporting Information). Each microchannel had a square cross-section with a width and height of 500 µm. The stage collecting the extruded sheet moved with a linear velocity of 300 mm/min. Figure 3a illustrates the extrusion of a hydrogel sheet.

Since the hydrogels with C_a-CNF_ of 0.50 and 1.0 wt.% exhibited a higher Young’s modulus and a more defined fibrillar structure than the hydrogels with C_a-CNF_ = 0.33 wt.%, the corresponding ink precursor was selected for extruding structurally anisotropic hydrogel sheets from the microfluidic printhead. A mixed a-CNF/gelatin suspension was extruded through the microfluidic printhead at room temperature at the volumetric flow rate, Q, varying from 5.0 to 9.0 mL/min (Figure S5, Supporting Information). In the printhead, the stream of a-CNF/gelatin mixture was split into 16 streams that merged at the outlet to form a sheet. During extrusion through the microchannels, the a-CNFs experienced shear stress that caused their orientation along the direction of extrusion. Owing to the variation in the shear stress within the microchannel (where the shear stress gradually decreases from the channel walls to the center and corners) [40], the extruded hydrogel sheets contained alternating regions of strong and weak a-CNF orientation (Figure 3a). The Brownian motion could change the orientation of a-CNFs [43]; however, extrusion-mediated a-CNF alignment in the direction of extrusion was preserved due to the covalent crosslinking of a-CNFs and gelatin in the extruded sheet. Figure S6, Supporting Information shows a photograph of the resulting smooth and weakly translucent hydrogel sheet.

Figure 3b shows that polarizing optical microscopic (POM) images of extruded hydrogel sheets exhibited periodic bright and dark stripes (corresponding to regions with strong and weak birefringence, respectively), which ran parallel to the direction of extrusion and correlated with the geometry of microchannels in the printhead [39,40]. The 560 ± 50 μm-wide bright regions originated from the microchannel walls, where the ink experienced a maximum shear stress [40]. A cast hydrogel used as a control did not exhibit a periodic variation in birefringence (Figure S7, Supporting Information). To confirm that the variation in the a-CNF alignment was not associated with spatial variation in hydrogel composition, we extruded hydrogel sheets from the mixture of a-CNFs and gelatin that were covalently labelled with distinct fluorescent dyes; that is, rhodamine B and fluorescein isothiocyanate (FITC), respectively. The fluorescence images revealed that a-CNFs and gelatin were homogeneously distributed throughout the hydrogel sheet (Figure S8, Supporting Information).

A comparison of representative POM images of hydrogels extruded at Q of 5.0 and 9.0 mL/min at C_a-CNF_ = 0.50 wt.% (Figure 3b, top two images) revealed a more pronounced periodic birefringence pattern for the hydrogel extruded at a higher volumetric flow rate. A similar effect was observed for the hydrogels with C_a-CNF_ increasing from 0.50 to 1.0 wt.%, both extruded at Q = 9.0 mL/min (Figure 3b, top right and bottom left images). The effect of C_a-CNF_ and Q value on the modulation in birefringence intensity was quantified by plotting the greyscale value profile across the sheet, that is, perpendicular to the extrusion direction. Figure 3b (bottom right) shows that as the value of Q increased from 5.0 to 9.0 mL/min (C_a-CNF_ = 0.50 wt.%), the intensity of birefringence in the bright regions (normalized to the intensity of light passing through the dark regions) increased by 38%. With C_a-CNF_ increasing from 0.50 to 1.0 wt.% (Q = 5.0 mL/min), the intensity of birefringence in the maxima peaks increased by 14%. Thus, control of hydrogel anisotropy could be achieved by varying C_a-CNF_ or Q, or both.

Notably, the hydrogel sheets retained the striped pattern of alternating bright and dark regions after 4-day incubation in HBSS at 37 °C (Figure S9, Supporting Information). For the hydrogel sheet with C_a-CNF_ = 1.0 wt.% extruded at Q = 9.0 mL/min, there was only a 2% difference in the intensity of the birefringence in the maxima peaks on Day 0 and Day 4; that is, no significant change in birefringence occurred over time. Thus, the shear-induced alignment of a-CNFs was preserved over 4 days in HBSS, owing to the formation of imine crosslinks in the hydrogel. This feature enabled the hydrogel use for long-term cell culture. Notably, while imine crosslinks are reported to degrade via hydrolysis in acidic conditions [44], this degradation should not take place at the neutral pH values used for cell culture.

Figure 3c shows representative SEM images of the regions of weak and strong structural anisotropy (corresponding to dark and bright regions, respectively, in POM images) in extruded hydrogel sheets. In Figure 3c (left), fibers were arranged randomly, while in Figure 3c (right), the fibers were oriented parallel to the direction of extrusion.

We note that due to the difference in velocity of the extruded ink and the movement of the stage during extrusion, some of the CNFs could lose their alignment along the direction of extrusion and orient perpendicular to the direction of extrusion as a result of retardation [45,46]. Nevertheless, this effect was not detrimental to the overall structural anisotropy in the hydrogel, as shown in POM and SEM images.

### 2.4. Structural Anisotropy of Extruded Hydrogel Threads

**Figure 4:**
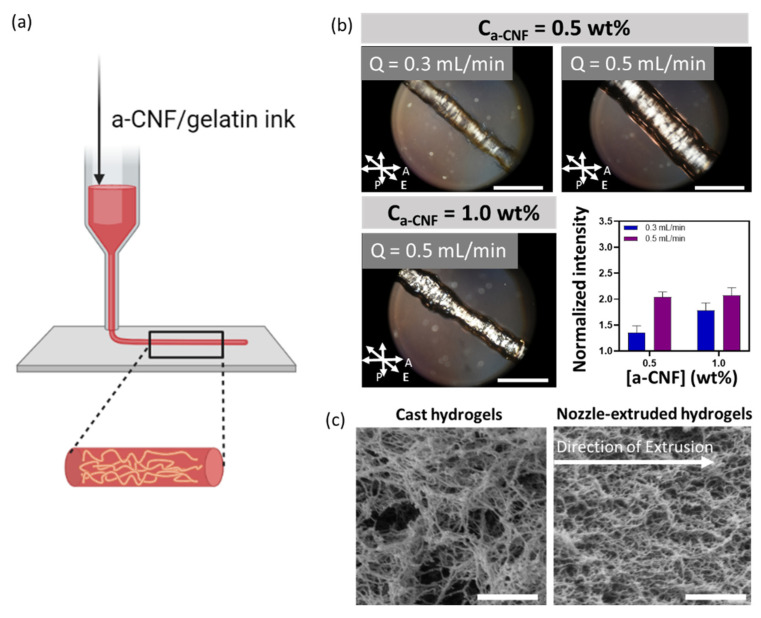
Structurally anisotropic hydrogel threads extruded through a nozzle. (**a**) Schematic depicting printing of hydrogel threads. (**b**) POM images of the extruded hydrogel threads at C_gelatin_ = 4.0 wt.%. Top left: C_a-CNF_ = 0.50 wt.%, Q = 0.3 mL/min. Top right: C_a-CNF_ = 0.50 wt.%, Q = 0.5 mL/min. Bottom left: C_a-CNF_ = 1.0 wt.%, Q = 0.5 mL/min. Scale bars are 2 mm. Bottom right: Average light intensity of the extruded hydrogel thread, normalized to that of the cast hydrogel with the same composition. Each bar represents three independently examined hydrogels. The arrows in the image depict the direction of extrusion (E) and the direction of the polarizer (P) and analyzer (A) with respect to the direction of extrusion. (**c**) SEM images of the cast (left) and extruded hydrogel threads (right). The gel was extruded at Q = 0.5 mL/min. C_a-CNF_ = 1.0 wt.%; C_gelatin_ = 4.0 wt.%. Scale bars are 1 μm.

In the second approach, we extruded the a-CNF/gelatin ink precursor thread using a 22G nozzle printhead, as shown in Figure 4a. The hydrogel ink with C_a-CNF_ of 0.50 and 1.0 wt.% were both tested with two extrusion flow rates, Q of 0.3 and 0.5 mL/min. To ensure that these flow rates were comparable to the flow rates in a single microchannel of the microfluidic printhead, the flow rates used for extrusion through the nozzle printhead were 16 times lower than the flow rates used in the microfluidic printhead extrusion.

Figure 4b revealed the anisotropic structure in the extruded hydrogel threads. A POM image of the thread extruded at Q = 0.5 mL/min had a brighter birefringence than that in the thread at the flow rate of 0.3 mL/min (50% and 16% increase for hydrogels of C_a-CNF_ = 0.50 and 1.0 wt.%), indicating a higher degree of CNF alignment in the thread extruded at a higher Q. This is due to stronger shear stress imposed on a-CNFs during extrusion at a higher flow rate [26]. As was the case with the extruded hydrogel sheets, the intensity of the bright region increased 31% with the increase in C_a-CNF_ from 0.50 to 1.0 wt.% (Q = 0.3 mL/min).

The anisotropic structure of the hydrogel thread was also observed in the SEM images. In Figure 4c (left), a cast hydrogel exhibited random distribution of the a-CNF fibers, while the fibers of the hydrogel extruded through the nozzle were mostly orientated in a direction parallel to the direction of extrusion (Figure 4c, right).

### 2.5. Effect of Structurally Anisotropic Hydrogels on Cell Orientation

The biocompatibility of the extruded a-CNF/gelatin hydrogel for HDFs during 2D and 3D cell culture was examined prior to exploring the effect of structural anisotropy on cell orientation. When HDFs were seeded on top of the extruded hydrogel (2D cell culture), cell proliferation was observed in hydrogels with both compositions (C_a-CNF_ of 0.50 or 1.0 wt.%; C_gelatin_ = 4.0 wt.%), with a normalized metabolic activity higher than 1.0 on Day 6 (Figure S10a, Supporting Information). For HDFs encapsulated in the hydrogels (3D cell culture), high metabolic activity of >75% on Day 6 was observed for HDFs cultured in both hydrogels (Figure S10b). Given the comparable biocompatibility of the two hydrogels, the hydrogel with C_a-CNF_ = 1.0 wt.% and C_gelatin_ = 4.0 wt.% was selected to study the effect of structural anisotropy on cell orientation during 2D and 3D cell culture, due to the higher degree of fiber alignment in such gels.

**Figure 5:**
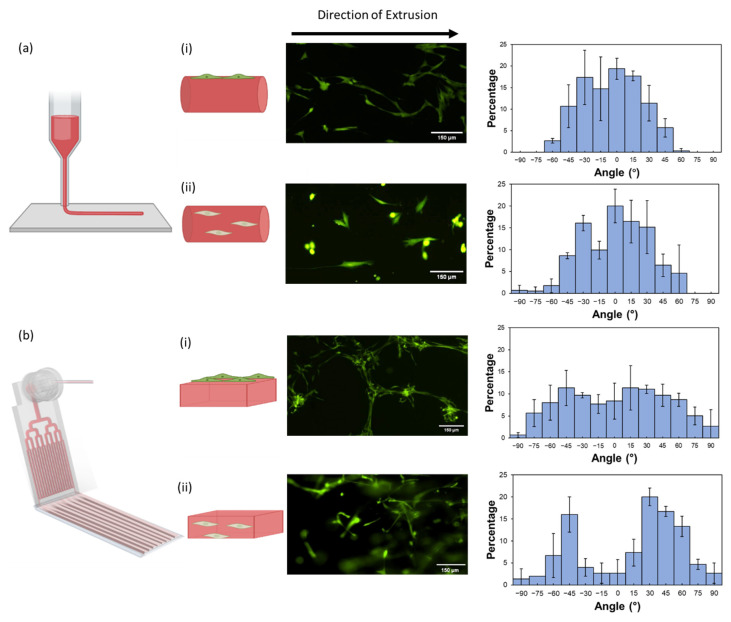
Characterization of HDF orientation during their 2D and 3D culture using extruded structurally anisotropic hydrogels. (**a**) Hydrogel sheets extruded from a nozzle printhead. (i) 2D culture of HDFs on the hydrogel threads on Day 1. (ii) 3D culture of HDFs encapsulated in the hydrogel threads on Day 6. (**b**) Hydrogel threads extruded from a microfluidic printhead. (i) 2D culture of HDFs on the hydrogel sheets on Day 6. (ii) 3D culture of HDFs encapsulated in the hydrogel sheets on Day 6. Middle column: fluorescence images of calcium-AM-stained HDFs cultured on or within the hydrogel. Right column: histograms of HDF cell orientation upon their culture on or within hydrogels on/in the hydrogels. Hydrogel composition: C_a-CNF_ = 1.0 wt.%, C_gelatin_ = 4.0 wt.%. Each error bar represents the standard deviation acquired in three independent samples.

After confirming the biocompatibility of the hydrogel, we first studied the effect of structural anisotropy on cell orientation with 2D cell culture on the hydrogels that were extruded using a microfluidic or a nozzle printhead. After extrusion, the hydrogels were soaked overnight in the culture medium at 37 °C. On Day 1, the HDFs were seeded on the hydrogel surface. Figure 5a(i) showed that one day after the seeding (Day 2), the HDFs adhered and spread on the surface of the hydrogel threads extruded from the nozzle printhead and preferentially aligned parallel to the direction of extrusion. The corresponding histogram revealed that >90% of the cells were oriented within ±45° from the direction of extrusion. To ensure that cell alignment on the hydrogel thread was not caused by the shape of the extruded hydrogel, the HDFs were seeded on a thread of a-CNF-free photo-crosslinked gelatin methacryloyl hydrogel extruded through the same nozzle printhead. In this chemically crosslinked structurally isotropic hydrogel, the orientation of HFDs on the hydrogel surface was random (Figure S11, Supporting Information).

In contrast, the HDFs seeded on the hydrogel sheets, extruded from the microfluidic printhead, did not spread out on the hydrogel surfaces until Day 6. In addition, Figure 5b(i) showed no clear evidence of cell alignment in the direction of extrusion. Instead, the histogram in this figure presented a broad distribution of the angle (from −90° to +90°) between the HDF orientation and the direction of extrusion.

We then explored the orientation of encapsulated HDFs with their 3D cell culture in the extruded hydrogels. For the hydrogel threads extruded through the nozzle, ~86% of HDFs encapsulated were orientated within ±45° to the direction of extrusion (Figure 5a(ii)). However, the HDFs encapsulated in the hydrogel sheets extruded through the microfluidic printhead did not exhibit a clear alignment along the direction of extrusion (Figure 5b(ii)). Instead, two unexpected peaks in the histogram at −45° and +30° were observed in the distribution of the cell orientation. We speculate that weak cell alignment in the hydrogel sheets extruded through the microfluidic printhead could be caused by the inhomogeneous distribution of the shear stress in the microchannels. During extrusion, the shear stress is concentrated mostly in the vicinity of the channel walls, while the majority of the ink in the channel experiences low shear stress (Figure S12a, Supporting Information). In the nozzle, however, the shear stress gradually and smoothly decreases from the wall to the center of the nozzle (Figure S12b, Supporting Information). There is a larger region in the cross-section of the nozzle that experiences high shear stress, compared to the microfluidic channel (the areas highlighted with red color in Figure S12, Supporting Information), which results in stronger and more uniform fiber alignment in the nozzle-extruded hydrogels. The results of simulations correlated with the distribution of birefringence intensity in the POM images (Figure 3b and Figure 4b), where the area of the bright region in the nozzle-extruded thread was, larger than that of one individual bright stripe in the microfluidically extruded hydrogel sheet.

Notably, the morphology of HDFs encapsulated in the hydrogel thread was different compared to the cells encapsulated in the hydrogel sheet. The HDFs encapsulated in the extruded hydrogel threads exhibited a larger spreading area, which potentially may indicate the transformation of the HDFs into myofibroblasts [47]. This effect agrees with the results of existing studies, in which hydrogel anisotropy facilitated the transformation of fibroblasts into myofibroblasts [6,48].

## 3. Conclusions

We developed a biocompatible fibrous hydrogel for studies of the impact of structural anisotropy on cell orientation. The hydrogel was formed by the covalent crosslinking between the aldehyde groups of CNFs and the amine groups of gelatin. This hydrogel showed >75% viability for both 2D and 3D culture with HDFs. A hydrogel precursor was used as an ink for extrusion-based 3D printing. The structural anisotropy of the hydrogel originated from the shear-induced a-CNF alignment in the direction of extrusion. The degree of gel anisotropy was varied by changing the composition of the ink and the extrusion flow rate. Extrusion was conducted using two different types of printheads, namely, a microfluidic printhead for sheet extrusion or a nozzle printhead for thread extrusion. It was established that the structural anisotropy of the hydrogel extruded through the nozzle induced the orientation of HDF cells along the direction of gel extrusion when they were cultured both on the gel surface and in the gel; that is, during 2D and 3D cultures, respectively. For HDFs seeded on and encapsulated in the extruded hydrogel threads, the alignment of 85% of the cells was at ≤45° to the direction of the extrusion. On the other hand, despite the existence of structural anisotropy, the a-CNF/gelatin hydrogel sheets extruded from the microfluidic printhead did not induce cell alignment during 2D and 3D HDF culture. This work presents a straightforward extrusion-based printing approach to a biocompatible fibrillar hydrogel with structural anisotropy, which leads to the preferential orientation of cells during their 2D and 3D culture.

## 4. Materials and Methods

### 4.1. Materials

Type A gelatin (300 g bloom), ethylene glycol (≥99% purity), sodium periodate (≥99% purity), 2,2,6,6-Tetramethyl-1-piperidinyloxy (TEMPO), sodium hypochlorite solution (NaClO, 10–15% active chlorine), and Rhodamine 123 were purchased from Sigma-Aldrich, Canada, and used without further purification. Sodium chlorite (NaClO_2_), sodium hydroxide aqueous solution (NaOH, 0.1 M), sodium phosphate and hydrochloric acid (HCl, 0.1 M) were used as received. A 3.0 wt.% aqueous suspension of CNFs was purchased from the University of Maine Process Development Center. Milli-Q deionized water was used (18.2 MΩ cm resistivity; Merck KGaA, Darmstadt, Germany) for all experiments. Dulbecco’s Modified Eagle Medium (DMEM), fetal bovine serum (FBS), penicillin/streptomycin, insulin, trypsin-EDTA solution, and Hank’s Balanced Salt Solution (HBSS, 1×) were supplied by Life Technologies (Thermo Fisher Scientific, Waltham, MA, USA). Dialysis tubing cellulose membrane (molecular weight cut-off 14,000, Sigma-Aldrich, St. Louis, MO, USA) was used for all dialysis experiments.

### 4.2. Modification of CNFs

An aqueous suspension (2 g, 67 mL) of CNFs was dispersed in 200 mL of deionized water containing 0.2 mmol TEMPO and 2 mmol sodium bromide in a 500 mL three-neck round-bottom flask. A total of 10 mmol sodium hypochlorite solution (6 mL) was dissolved in 20 mL of deionized water and subsequently added to the flask. The suspension was stirred at 500 rpm (46 g) at room temperature for 24 h, with the pH being adjusted to 10–10.5 using 0.5 M NaOH throughout the reaction. The reaction was quenched by adding ethylene glycol. The TEMPO-oxidized CNFs (TOCNFs) were purified by dialysis using Milli-Q water for 7 days [49].

Next, the TOCNFs were surface-oxidized using sodium periodate (NaIO_4_) to yield a-CNFs [50,51]. Sodium periodate and the TOCNF suspension were mixed at a mass ratio of 1:1 (NaIO_4_ (g): TOCNFs (g)). Aluminium foil was used to cover the flask to prevent NaIO_4_ from photodegradation. The NaIO_4_/TOCNFs mixture was continuously stirred at room temperature for 24 h and, subsequently, the reaction of aldehyde group oxidation was quenched by ethylene glycol. The a-CNF suspension was dialyzed against deionized water for 7 days, with replacement of water every 12 h, and subsequently concentrated by rotary evaporation. The concentrated a-CNFs suspension was stored at 4 °C for further use.

### 4.3. Characterization of a-CNFs

The presence of aldehyde groups on a-CNF was confirmed using attenuated total reflectance–Fourier transform infrared spectroscopy (ATR-FTIR, Perkin Elmer spectrum 100 FTIR Spectrometer with Universal ATR). Pristine CNFs were used as a control sample.

The molar concentration of aldehyde groups on the a-CNFs was determined by the conversion of aldehyde to oxime by titration. Briefly, a-CNFs (0.1 g) were added to 30 mL water and its pH was adjusted to 4.5 with 0.1 M HCl. Hydroxylamine hydrochloride (NH_2_OH·HCl) solution (0.5 g in 30 mL, pH 4.5) was added to the a-CNF suspension. The mixture was stirred at 300 rpm (17 g) at room temperature for 24 h. The conversion of aldehyde to oxime was characterized by titration and determined by calculating the consumption of 0.1 M NaOH that was used to neutralize excess HCl [52].

Transmission electron microscopy (TEM, Hitachi HT7700) was used to image pristine CNFs, TOCNFs and a-CNFs. A droplet of 0.001 wt.% suspension of pristine CNFs, TOCNFs and a-CNFs was pipetted onto a copper grid (Carbon 400 mesh; Electron Microscopy Sciences, Hatfield, PA, USA). The sample was allowed to dry at room temperature before imaging.

### 4.4. Preparation and Characterization of Hydrogels

An aqueous suspension of a-CNFs in HBSS was mixed with an aqueous solution of gelatin in different volumetric ratios to prepare hydrogels with a total a-CNF concentration from 0.25 to 1.0 wt.% and gelatin concentration of 4.0 wt.%.

The rheological properties of the a-CNF/gelatin mixture (gel precursor) were measured using a rheometer (DHR-1, TA Instruments Inc., New Castle, DE, USA) with a cone-plate geometry (cone diameter of 40 mm and an angle of 0.97°). An integrated Peltier plate was used to control the temperature and a solvent trap was used to prevent solvent evaporation during measurements. The storage modulus (G′) and loss modulus (G′′) of the hydrogel were characterized at 37 °C at 1% strain and 1 Hz frequency.

The compression Young’s modulus of the hydrogel was determined in cyclic compression experiments using a Mach-1 Mechanical tester (Biomomentum Inc., Quebec, Canada). To determine the Young’s modulus as a function of time, hydrogel disks (3 mm in height and 9 mm in diameter) were prepared at 22 °C and equilibrated at 37 °C for 24 and 72 h prior to compression measurements. The hydrogel disks were compressed to the 20% strain at a rate of 0.01 mm/s. The Young’s modulus of the hydrogels was calculated from the slope of the linear portion in the stress−strain curve.

SEM was used to characterize the morphology of a-CNF/gelatin hydrogels. Hydrogel samples were fixed with 2.5 wt.% glutaraldehyde and subsequently transferred into a 30% ethanol/water solution. The solvent was sequentially changed to 40, 50, 60, 70, 80, 90 and 100% of ethanol with at least 30 min incubation in each ethanol solution. The hydrogels were placed in an Autosamdri-810 Tousimis critical point dryer and the ethanol was exchanged with liquid CO_2_ for supercritical drying. The dried gels were subsequently fractured and gold-sputtered using a SC7640 High Resolution Sputter Coater (Quorum Technologies) for 45 s at 2.0 kV and 20 mA before SEM imaging using a Quanta FEI microscope.

### 4.5. Extrusion-Based Printing of Hydrogels

A microfluidic printhead was 3D-fabricated by stereolithography-based printing as described previously [39]. The nozzle printhead was a 22G blunt needle (inner diameter: 0.41 mm) purchased from CELLINK. The ink for extrusion-based printing was a mixture of the a-CNFs and gelatin. The concentration of a-CNFs in the ink varied from 0.50 to 1.0 wt.% and the concentration of gelatin in the ink was 4.0 wt.%. The ink was introduced into a syringe and incubated at 37 °C for 10 min, and subsequently maintained at room temperature for 45 min. The syringe loaded with the ink was assembled onto an infusion pump (PHD Ultra; Harvard Apparatus, Holliston, MA, USA). The printhead was connected to the syringe via a Luer lock connection and mounted to an in-house modified ENDER 3 3D printer. The printhead moving speed of the printhead along the platform was set to be 300 mm/min for the microfluidic printhead and 600 mm/min for the nozzle printhead and was controlled by the Pronterface software (http://www.pronterface.com, accessed on 1 September 2021). The volumetric flow rate, Q, of the ink during extrusion through the microfluidic printhead varied from 5.0 to 9.0 mL/min, and from 0.3 to 0.5 mL/min for extrusion through the nozzle printhead.

### 4.6. Measurement of Normalized Birefringence Light Intensity

POM images (Olympus BX51) were used to examine the shear-induced birefringence of extruded hydrogel sheets and threads as described previously [39,40,45]. POM imaging was carried out with cross-polarizers oriented at 45° with respect to the flow direction. A camera was used to capture images (6000 × 4000 pixel) with an exposure time at 1/13 sec and ISO speed of 800. ImageJ software (NIH) was used to characterize birefringence intensity. For the hydrogel sheets extruded from the microfluidic printhead, the normalized light intensity was calculated as the ratio of intensities of the light passing through the bright and the dark stripes in the extruded hydrogel sample (with cross-polarizer assembly at 45°). In the hydrogel threads extruded from the nozzle, the normalized light intensity was calculated as the ratio of intensities of light passing through the extruded thread and the cast hydrogel with the same composition.

### 4.7. Cell Culture

Human dermal fibroblasts (HDFs, ATCC) were cultured in DMEM with 10% FBS and 1% penicillin–streptomycin. The cells were cultured in T75 tissue culture flasks and incubated at 37 °C with 5% CO_2_. For cell passaging, the cells were first detached from the surface by using a 0.25 wt.% Trypsin-EDTA solution (GIBCO) and subsequently spun down at 184 g for 3 min. The supernatant was discarded and replaced by fresh cell culture medium to re-suspend the cells. Subsequently, 20 vol.% of the cell suspension was transferred to a new tissue culture flask with the fresh medium.

### 4.8. Seeding HDFs on the Printed Hydrogels

Prior to seeding HDFs for 2D culture, the hydrogels were extruded with either a microfluidic printhead, or a nozzle printhead. The ink with a-CNF and gelatin concentrations of 1.0 and 4.0 wt.%, respectively, was extruded at a volumetric flow rate of 9.0 or 0.5 mL/min through the microfluidic or nozzle printhead, respectively. The extruded hydrogels were exposed to the air for 1 h at room temperature and subsequently, submerged in the cell culture medium at room temperature for 4 h. After that, the hydrogels submerged in the cell culture medium were incubated overnight at 37 °C with a constant supply of 5% CO_2_. On Day 2, HDFs were seeded on top of the hydrogels at a density of 31,250 cells/cm^2^. The orientation of cells on the extruded threads or sheets was examined on Day 1 and Day 6 after seeding, respectively.

### 4.9. Extrusion-Based Printing of Cell-Laden Hydrogels

The HDF cells were washed with HBSS, trypsinized (0.25 wt.% trypsin-EDTA solution) and mixed with an aqueous CNF/gelatin suspension. The cell density and the concentration of a-CNFs and gelatin in the mixture was 500 cell/μL, 1.0 and 4.0 wt.%, respectively. The cell-laden hydrogels were extruded at a volumetric flow rate of 9.0 mL/min (through a microfluidic printhead) and of 0.5 mL/min (through a nozzle printhead). The extruded hydrogels were exposed to the air for 1 h at room temperature and then submerged in the cell culture medium at room temperature for 4 h. Subsequently, the hydrogels in the culture medium were incubated at 37 °C with a constant supply of 5% CO_2_ and the medium being changed every 3 days. The orientation of cells was analyzed on Day 6.

### 4.10. Analysis of Cell Orientation

An inverted fluorescent microscope (Nikon Eclipse-Ti) was used to image the HDFs seeded on or encapsulated in the extruded hydrogel. The HDFs were stained with Calcein AM viability dye (Invitrogen™, Waltham, MA, USA) and imaged under the FITC channel (excitation at 490 nm and emission at 525 nm) of the fluorescence microscope. Cell orientation in the structurally anisotropic regions was analyzed using ImageJ software by measuring the angle between the direction of cell spreading and the direction of extrusion (the latter direction was taken as 0°). This angle could vary from −90° to +90°. For the hydrogel sheets extruded from the microfluidic printhead, POM images were overlayed on top of fluorescence images, in order to locate the regions with structural anisotropy. For each system, cell orientation was analyzed in three regions in three independently extruded hydrogels and was represented as a histogram. The bins in the histogram represented the averaged relative frequency (%) of the corresponding cell orientation and error bars represented the standard deviation. In 2D culture experiments, the orientation of 100 HDFs was measured for each region. In 3D culture experiments, the orientation of 50 HDFs was measured for each region.

## Data Availability

Data available on request.

## Supplementary Materials

The following supporting information can be downloaded at: https://www.mdpi.com/article/10.3390/gels8110685/s1, Figure S1: Characterization of TOCNFs; Figure S2: ATR-FTIR of pristine CNF and a-CNF; Figure S3: Rheological characterization of gelation of a-CNF/gelatin precursor at 37℃; Figure S4: Dimensions and design of the microfluidic printhead; Figure S5: Microfluidic printing set-up for extrusion of hydrogel sheets; Figure S6: Representative image of an extruded hydrogel sheet; Figure S7: POM images of cast and extruded hydrogels; Figure S8: Fluorescence images of FITC-labelled gelatin and rhodamine-labelled a-CNFs in the hydrogel sheet; Figure S9: POM images of extruded hydrogels over time; Figure S10: Overtime changes of metabolic activity of HDFs; Figure S11: Characterization of HDF orientation on the CNF-free photo-crosslinked gelatin methacryloyl hydrogels; Figure S12: Simulation of the shear stress distribution on the a-CNF/gelatin ink during extrusion. Table S1: Quantification of number of aldehyde groups on a-CNF.

## References

[1] Khuu N., Kheiri S., Kumacheva E. (2021). Structurally anisotropic hydrogels for tissue engineering. Trends Chem..

[2] Beldjilali-Labro M., Garcia Garcia A., Farhat F., Bedoui F., Grosset J.-F., Dufresne M., Legallais C. (2018). Biomaterials in Tendon and Skeletal Muscle Tissue Engineering: Current Trends and Challenges. Materials.

[3] Magnusson S.P., Heinemeier K.M., Kjaer M. (2016). Collagen homeostasis and metabolism. Metab. Influ. Risk Tendon Disord..

[4] Ziv V., Wagner H.D., Weiner S. (1996). Microstructure-microhardness relations in parallel-fibered and lamellar bone. Bone.

[5] Saffitz J.E., Kanter H.L., Green K.G., Tolley T.K., Beyer E.C. (1994). Tissue-specific determinants of anisotropic conduction velocity in canine atrial and ventricular myocardium. Circ. Res..

[6] Li Y., Xiao Z., Zhou Y., Zheng S., An Y., Huang W., He H., Yang Y., Li S., Chen Y. (2019). Controlling the multiscale network structure of fibers to stimulate wound matrix rebuilding by fibroblast differentiation. ACS Appl. Mater. Interfaces.

[7] Huang C., Fu X., Liu J., Qi Y., Li S., Wang H. (2012). The involvement of integrin β1 signaling in the migration and myofibroblastic differentiation of skin fibroblasts on anisotropic collagen-containing nanofibers. Biomaterials.

[8] Meek K.M., Knupp C. (2015). Corneal structure and transparency. Prog. Retin. Eye Res..

[9] Shah A., Brugnano J., Sun S., Vase A., Orwin E. (2008). The Development of a Tissue-Engineered Cornea: Biomaterials and Culture Methods. Pediatr. Res..

[10] Changoor A., Nelea M., Méthot S., Tran-Khanh N., Chevrier A., Restrepo A., Shive M.S., Hoemann C.D., Buschmann M.D. (2011). Structural characteristics of the collagen network in human normal, degraded and repair articular cartilages observed in polarized light and scanning electron microscopies. Osteoarthr. Cartil..

[11] Fratzl P., Weinkamer R. (2007). Nature’s hierarchical materials. Prog. Mater. Sci..

[12] Ueda M., Saito S., Murata T., Hirano T., Bise R., Kabashima K., Suzuki S. (2019). Combined multiphoton imaging and biaxial tissue extension for quantitative analysis of geometric fiber organization in human reticular dermis. Sci. Rep..

[13] Ottenio M., Tran D., Ní Annaidh A., Gilchrist M.D., Bruyère K. (2015). Strain rate and anisotropy effects on the tensile failure characteristics of human skin. J. Mech. Behav. Biomed. Mater..

[14] Lynch H.A., Johannessen W., Wu J.P., Jaw A., Elliott D.M. (2003). Effect of Fiber Orientation and Strain Rate on the Nonlinear Uniaxial Tensile Material Properties of Tendon. J. Biomech. Eng..

[15] Weiner S., Wagner H.D. (1998). The material bone: Structure-mechanical function relations. Annu. Rev. Mater. Sci..

[16] Caliari S.R., Burdick J.A. (2016). A practical guide to hydrogels for cell culture. Nat. Methods.

[17] Shin S., Hyun J. (2021). Rheological properties of cellulose nanofiber hydrogel for high-fidelity 3D printing. Carbohydr. Polym..

[18] Shin S., Park S., Park M., Jeong E., Na K., Youn H.J., Hyun J. (2017). Cellulose Nanofibers for the Enhancement of Printability of Low Viscosity Gelatin Derivatives. BioResources.

[19] De France K.J., Hoare T., Cranston E.D. (2017). Review of Hydrogels and Aerogels Containing Nanocellulose. Chem. Mater..

[20] Sanandiya N.D., Vasudevan J., Das R., Lim C.T., Fernandez J.G. (2019). Stimuli-responsive injectable cellulose thixogel for cell encapsulation. Int. J. Biol. Macromol..

[21] Apelgren P., Karabulut E., Amoroso M., Mantas A., Martínez Ávila H., Kölby L., Kondo T., Toriz G., Gatenholm P. (2019). In Vivo Human Cartilage Formation in Three-Dimensional Bioprinted Constructs with a Novel Bacterial Nanocellulose Bioink. ACS Biomater. Sci. Eng..

[22] Chen R.-D., Huang C.-F., Hsu S.-h. (2019). Composites of waterborne polyurethane and cellulose nanofibers for 3D printing and bioapplications. Carbohydr. Polym..

[23] Kim H.J., Oh D.X., Choy S., Nguyen H.-L., Cha H.J., Hwang D.S. (2018). 3D cellulose nanofiber scaffold with homogeneous cell population and long-term proliferation. Cellulose.

[24] Prince E., Kumacheva E. (2019). Design and applications of man-made biomimetic fibrillar hydrogels. Nat. Rev. Mater..

[25] Kumbar S., James R., Nukavarapu S., Laurencin C. (2008). Electrospun nanofiber scaffolds: Engineering soft tissues. Biomed. Mater..

[26] Li K., Clarkson C.M., Wang L., Liu Y., Lamm M., Pang Z., Zhou Y., Qian J., Tajvidi M., Gardner D.J. (2021). Alignment of Cellulose Nanofibers: Harnessing Nanoscale Properties to Macroscale Benefits. ACS Nano.

[27] Wang C., Pan Z.-Z., Lv W., Liu B., Wei J., Lv X., Luo Y., Nishihara H., Yang Q.-H. (2019). A Directional Strain Sensor Based on Anisotropic Microhoneycomb Cellulose Nanofiber-Carbon Nanotube Hybrid Aerogels Prepared by Unidirectional Freeze Drying. Small.

[28] Kim H.C., Kim J.W., Zhai L., Kim J. (2019). Strong and tough long cellulose fibers made by aligning cellulose nanofibers under magnetic and electric fields. Cellulose.

[29] Rezaei A., Nasirpour A., Fathi M. (2015). Application of Cellulosic Nanofibers in Food Science Using Electrospinning and Its Potential Risk. Compr. Rev. Food Sci. Food Saf..

[30] Eom S., Park S.M., Hong H., Kwon J., Oh S.-R., Kim J., Kim D.S. (2020). Hydrogel-Assisted Electrospinning for Fabrication of a 3D Complex Tailored Nanofiber Macrostructure. ACS Appl. Mater. Interfaces.

[31] Huang L., Du X., Fan S., Yang G., Shao H., Li D., Cao C., Zhu Y., Zhu M., Zhang Y. (2019). Bacterial cellulose nanofibers promote stress and fidelity of 3D-printed silk based hydrogel scaffold with hierarchical pores. Carbohydr. Polym..

[32] Sydney Gladman A., Matsumoto E.A., Nuzzo R.G., Mahadevan L., Lewis J.A. (2016). Biomimetic 4D printing. Nat. Mater..

[33] Kim T., Bao C., Hausmann M., Siqueira G., Zimmermann T., Kim W.S. (2019). 3D Printed Disposable Wireless Ion Sensors with Biocompatible Cellulose Composites. Adv. Electron. Mater..

[34] Isogai A., Saito T., Fukuzumi H. (2011). TEMPO-oxidized cellulose nanofibers. Nanoscale.

[35] Prince E., Alizadehgiashi M., Campbell M., Khuu N., Albulescu A., De France K., Ratkov D., Li Y., Hoare T., Kumacheva E. (2018). Patterning of Structurally Anisotropic Composite Hydrogel Sheets. Biomacromolecules.

[36] Xue Y., Mou Z., Xiao H. (2017). Nanocellulose as a sustainable biomass material: Structure, properties, present status and future prospects in biomedical applications. Nanoscale.

[37] Klemm D., Kramer F., Moritz S., Lindström T., Ankerfors M., Gray D., Dorris A. (2011). Nanocelluloses: A New Family of Nature-Based Materials. Angew. Chem. Int. Ed..

[38] Winter H.H., Chambon F. (1986). Analysis of Linear Viscoelasticity of a Crosslinking Polymer at the Gel Point. J. Rheol..

[39] Khuu N., Alizadehgiashi M., Gevorkian A., Galati E., Yan N., Kumacheva E. (2019). Temperature-Mediated Microfluidic Extrusion of Structurally Anisotropic Hydrogels. Adv. Mater. Technol..

[40] Gevorkian A., Morozova S.M., Kheiri S., Khuu N., Chen H., Young E., Yan N., Kumacheva E. (2021). Actuation of Three-Dimensional-Printed Nanocolloidal Hydrogel with Structural Anisotropy. Adv. Funct. Mater..

[41] Alizadehgiashi M., Gevorkian A., Tebbe M., Seo M., Prince E., Kumacheva E. (2018). 3D-Printed Microfluidic Devices for Materials Science. Adv. Mater. Technol..

[42] Hansen C.J., Saksena R., Kolesky D.B., Vericella J.J., Kranz S.J., Muldowney G.P., Christensen K.T., Lewis J.A. (2013). High-Throughput Printing via Microvascular Multinozzle Arrays. Adv. Mater..

[43] Mittal N., Ansari F., Gowda V.K., Brouzet C., Chen P., Larsson P.T., Roth S.V., Lundell F., Wågberg L., Kotov N.A. (2018). Multiscale Control of Nanocellulose Assembly: Transferring Remarkable Nanoscale Fibril Mechanics to Macroscale Fibers. ACS Nano.

[44] Zhang Y., Tao L., Li S., Wei Y. (2011). Synthesis of Multiresponsive and Dynamic Chitosan-Based Hydrogels for Controlled Release of Bioactive Molecules. Biomacromolecules.

[45] Park H., Lee K.H., Kim Y.B., Ambade S.B., Noh S.H., Eom W., Hwang J.Y., Lee W.J., Huang J., Han T.H. (2018). Dynamic assembly of liquid crystalline graphene oxide gel fibers for ion transport. Sci. Adv..

[46] Trebbin M., Steinhauser D., Perlich J., Buffet A., Roth S.V., Zimmermann W., Thiele J., Förster S. (2013). Anisotropic particles align perpendicular to the flow direction in narrow microchannels. Proc. Natl. Acad. Sci..

[47] Hinz B., Phan S.H., Thannickal V.J., Prunotto M., Desmoulière A., Varga J., De Wever O., Mareel M., Gabbiani G. (2012). Recent developments in myofibroblast biology: Paradigms for connective tissue remodeling. Am. J. Pathol..

[48] Echave M.C., Domingues R.M.A., Gómez-Florit M., Pedraz J.L., Reis R.L., Orive G., Gomes M.E. (2019). Biphasic Hydrogels Integrating Mineralized and Anisotropic Features for Interfacial Tissue Engineering. ACS Appl. Mater. Interfaces.

[49] Saito T., Hirota M., Tamura N., Kimura S., Fukuzumi H., Heux L., Isogai A. (2009). Individualization of Nano-Sized Plant Cellulose Fibrils by Direct Surface Carboxylation Using TEMPO Catalyst under Neutral Conditions. Biomacromolecules.

[50] Varma A.J., Kulkarni M.P. (2002). Oxidation of cellulose under controlled conditions. Polym. Degrad. Stab..

[51] Kumari S., Ram B., Kumar D., Ranote S., Chauhan G.S. (2018). Nanoparticles of oxidized-cellulose synthesized by green method. Mater. Sci. Energy Technol..

[52] Kim U.-J., Wada M., Kuga S. (2004). Solubilization of dialdehyde cellulose by hot water. Carbohydr. Polym..

