# Supplementary material for: Printing Structurally Anisotropic Biocompatible Fibrillar Hydrogel for Guided Cell Alignment
Source: Gels. 2022 Oct 22;8(11):685. doi: 10.3390/gels8110685 (PMC9689575; doi:10.3390/gels8110685)
Supplement: Supplementary file 1 [file gels-08-00685-s001.zip › gels-1943850-supplementary.pdf]

Supplementary Materials: Printing Structurally Anisotropic Biocompatible Fibrillar Hydrogel for Guided Cell Alignment

Zhengkun Chen [1,†], Nancy Khuu [1,†], Fei Xu [1], Sina Kheiri [2], Ilya Yakavets [1], Faeze Rakhshani [1], Sofia Morozova [1,3]
and Eugenia Kumacheva [1,4,5,*]

[1] Department of Chemistry, University of Toronto, Toronto, ON M5S 3H6, Canada
[2] Department of Mechanical and Industrial Engineering, University of Toronto, Toronto, ON M5S 3G8, Canada
[3] N.E. Bauman Moscow State Technical University, 5/1 2nd Baumanskaya Street, 105005 Moscow, Russia
[4] Department of Chemical Engineering and Applied Chemistry, University of Toronto, Toronto, ON M5S 3E5, Canada
[5] The Institute of Biomedical Engineering, University of Toronto, Toronto, ON M5S 3G9, Canada
* Correspondence: eugenia.kumacheva@utoronto.ca; Tel.: +1-416-978-3576
† These authors contributed equally to this work.

S1. Characterization of TOCNF Suspension

Figure S1a shows the transmittance of the suspension of pristine CNFs and TOCNFs measured at different wavelengths in ultraviolet-visible (UV-vis) spectroscopy (path length of cuvette = 10 mm). For a 0.1 wt.% suspension of CNFs, following TEMPO modification and the dissociation of CNF bundles, the transmittance increased from 4.3 to 78.1% at 400 nm and from 11.4 to 87.8% at 600 nm. Figure S1b shows the distribution of diameters of TOCNFs. Following TEMPO modification, the average diameter of TOCNFs was 13.8 ± 2.4 nm.

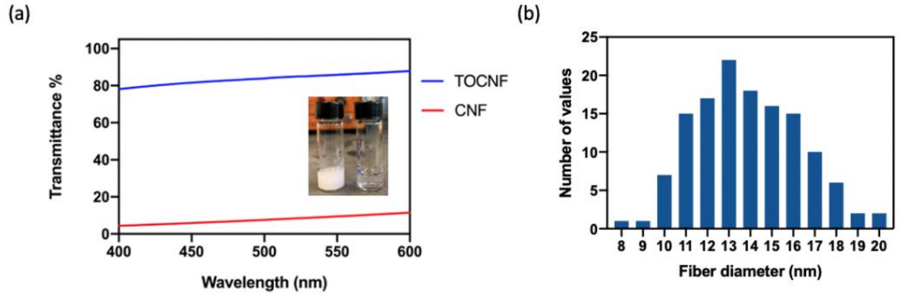

Figure S1. Characterization of TOCNFs. (a) The transmittance of pristine CNFs and TOCNFs.

The inset image depicts 1.0 wt.% suspension of pristine CNFs (left) and TOCNFs (right). (b) Histogram of the distribution of diameters of TOCNFs.

S2. Characterization of a-CNF Suspension

The modification of TOCNFs with sodium periodate was used to introduce aldehyde groups on the TOCNFs, thus, forming a-CNFs. Figure S2 shows the infrared spectrum for pristine CNFs and a-CNFs. A peak at 1700 cm^{-1} corresponding to the carbonyl stretch of the aldehyde groups was observed in the spectrum, confirming the functionalization of the TOCNF surface.

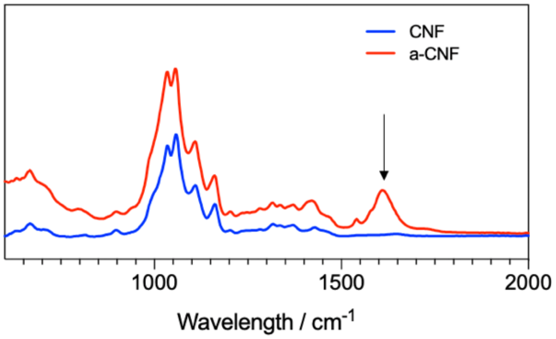

Figure S2. ATR-FTIR of pristine CNFs and a-CNFs.

Table S1 depicts the molar concentration of aldehyde groups on a-CNFs, determined by titration with hydroxylamine hydrochloride. The molar concentration of the surface aldehyde groups varied as a function of the reaction time and TOCNF: $NaIO_4$ mass ratio. For a fixed mass ratio of TOCNF: $NaOIO_4$, the concentration of aldehyde groups increased with a longer reaction time. For a particular reaction time, the concentration of aldehyde groups increased with a greater TOCNF: $NaOIO_4$ mass ratio.

Table S1. Quantification of the number of aldehyde groups on a-CNF as determined by titration with hydroxylamine hydrochloride.

TOCNF : $NaIO_4$	Reaction time (h)	-CHO (mmol) per g CNF
1:1	4	1.95
1:1	24	2.60
1:2	4	2.16
1:2	24	3.36

S3. Rheology of the a-CNF/Gelatin Mixture

Figure S3 shows the variation in the storage modulus, G′, and loss modulus, G′′, examined in the rheology experiments for suspensions with $C_{a\text{-}CNF}$ of 0.25, 0.33, and 1.0 wt.% at $C_{Gelatin}$ = 4.0 wt.%. Gelation was not observed for mixtures with $C_{a\text{-}CNF}$ = 0.25 and $C_{Gelatin}$ = 4.0 wt.% after 8 h, due to a limited number of aldehyde groups on the TOCNF surface and the insufficient density of Schiff-base crosslinks. For mixtures with $C_{a\text{-}CNF}$ of 0.33 wt.% and 1.0 wt.% at $C_{Gelatin}$ = 4.0 wt.%, gelation was observed at 5200s and immediately after mixing a-CNFs and gelatin, respectively.

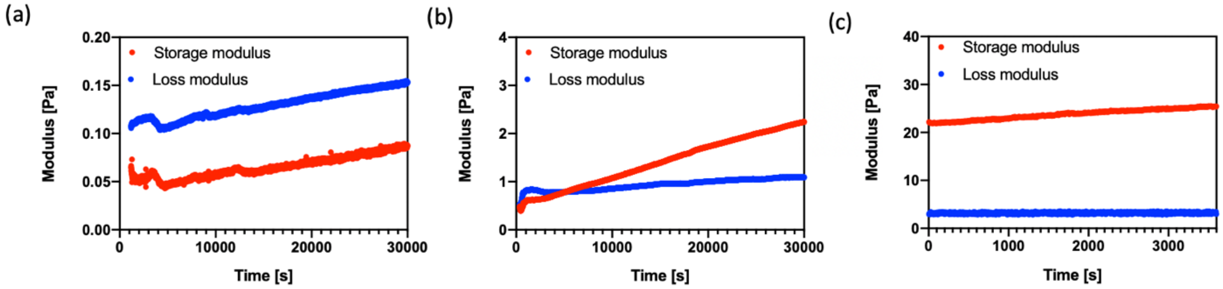

Figure S3. Rheological characterization of gelation of a-CNF/gelatin precursor mixture at 37 °C, depicting the storage modulus (red) and loss modulus (blue). The $C_{a\text{-}CNF}$ in the hydrogel was 0.25 wt.% (a), 0.33 wt.% (b), and 1.0 wt.% (c). $C_{Gelatin}$= 4.0 wt.%. The ratio of molar concentrations of aldehyde groups on a-CNFs to amine groups on gelatin was 0.75: 1 (a), 1:1 (b) and 3: 1 (c).

S4. Design of the Microfluidic Printhead Device

Figure S4 illustrates the design and dimensions of the microfluidic printhead used for the extrusion of a-CNF/gelatin hydrogel sheets. The a-CNF/gelatin ink is introduced into the inlet and bifurcated into 4 generations of parallel microchannels, where the number of channels in each generation is 2.

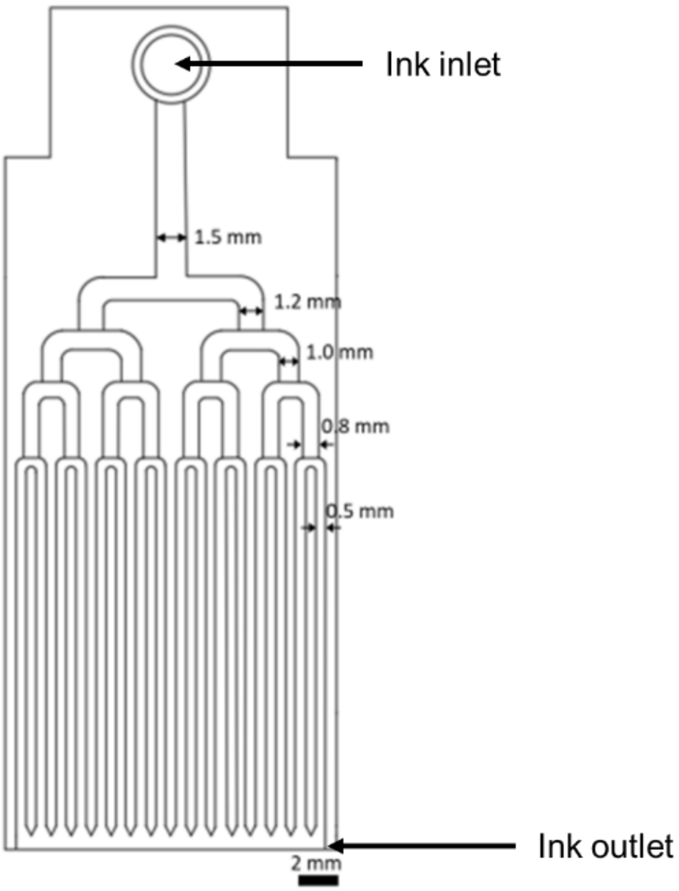

Figure S4. Design and dimensions of the microfluidic printhead.

S5. Microfluidic Printing Setup

Figure S5 depicts a photograph of the 3D extrusion-based printing setup for the fabrication of an a-CNF/gelatin hydrogel sheet. A 3D-printed microfluidic printhead was mounted on a 3D printer, with a stage moving with a linear velocity of 300 mm/min. The hydrogel sheet was extruded into an in-house made sample holder, consisting of a PDMS chamber on top of a glass slide.

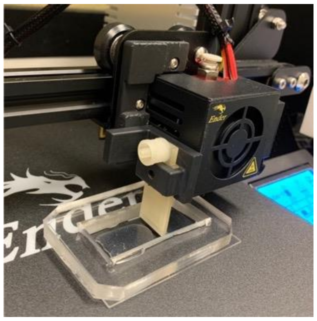

Figure S5. Microfluidic printing set-up for extrusion of hydrogel sheets.

S6. Representative Photograph of an Extruded Hydrogel Sheet

Figure S6 shows a photograph of the extruded hydrogel sheet, which appeared smooth and translucent.

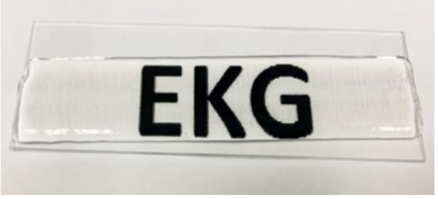

Figure S6. Representative image of an extruded hydrogel sheet. The hydrogel was extruded on a glass slide at a flow rate of 9 mL/min with $C_{a\text{-}CNF}$ = 1.0 wt.% and $C_{Gelatin}$= 4.0 wt.%.

S7. Measurement of Birefringence in Extruded HYDROGEL sheets

Figure S7a and b show representative POM images of the cast and extruded hydrogel sheets. While the cast gel appeared mostly dark, the extruded hydrogel sheet exhibited a periodic variation in birefringence, which correlated with microchannel geometry in the microfluidic printhead. Thus, the anisotropy of the extruded hydrogel sheet originated from the extrusion of the a-CNF/hydrogel ink. Figure S7c shows the corresponding grey value profile for the extruded hydrogel sheet in Figure S7b.

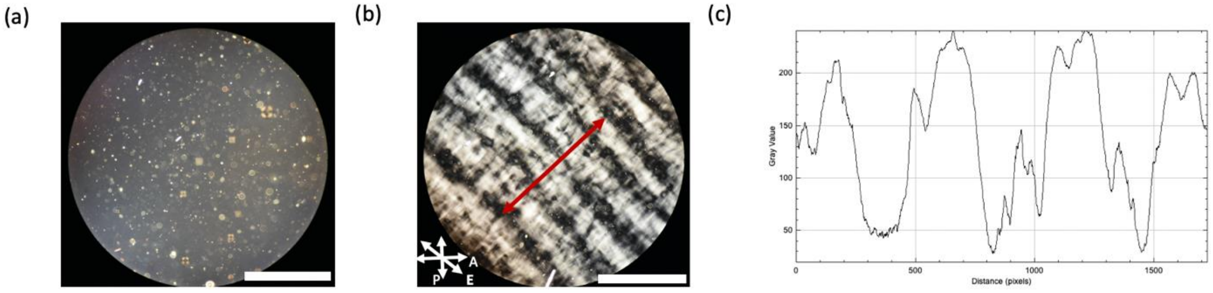

Figure S7. POM images of the cast and extruded hydrogels. (a) POM image of a cast hydrogel. (b) POM image of an extruded hydrogel sheet with $C_{a\text{-}CNF}$ = 1.0 wt.% and $C_{Gelatin}$= 4.0 wt.%, extruded at Q = 9 mL/min. The red arrow indicates the line, perpendicular to the direction of extrusion, used to measure the grey scale value across the hydrogel sheet. The white arrows in the image depict the direction of extrusion (E) and the direction of the polarizer (P) and analyzer

(A) with respect to the direction of extrusion. (c) The corresponding grey value profile across the extruded hydrogel sheet. Scale bars are 2 mm.

S8. Distribution of a-CNF and Gelatin in the Extruded Hydrogel Sheet

Figure S8 shows representative fluorescence images of hydrogel sheets extruded from fluorescently labelled a-CNF and gelatin. Gelatin was covalently labelled with fluorescein isothiocyanate (FITC, green emission) and a-CNF was covalently labelled with rhodamine B (red). Both gelatin (Figure S8, left) and a-CNF (Figure S8, right) were uniformly distributed throughout the entire hydrogel sheet. Thus, the variation in birefringence intensity was not caused by a spatial variation in polymer compositions.

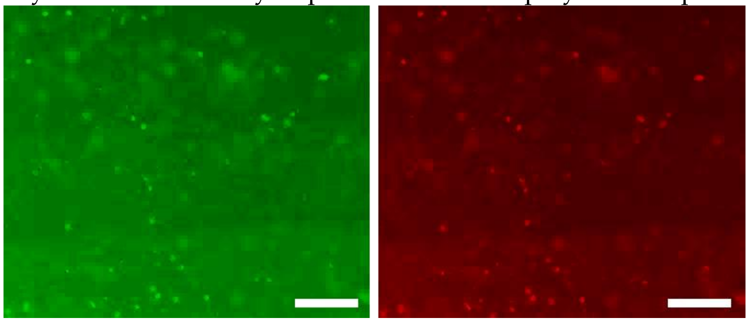

Figure S8. Fluorescence images of the a-CNF/gelatin hydrogel. The gel contains FITC-labelled gelatin (left) and rhodamine B-labelled a-CNFs (right). The hydrogel sheet was extruded at Q = 9 mL/min. $C_{a\text{-}CNF}$ = 0.5 wt.%, $C_{Gelatin}$= 4 wt.%. Scale bars are 500 μm.

S9. Measurement of Birefringence of Extruded Hydrogel Sheets over Time

Figure S9a and b show representative POM images of the hydrogel sheet with $C_{a\text{-}CNF}$ = 1.0 wt.% and $C_{Gelatin}$= 4.0 wt.% immediately after extrusion (day 0) and after 4-day incubation in HBSS at 37 ºC. Both Figure S9a and b exhibited a birefringence pattern with alternating bright and dark regions. Figure S9c depicts the temporal change in the average normalized light intensity over time. The average normalized light intensity on Day 0 and Day 4 was 1.90 ± 0.1 and 1.86 ± 0.06, respectively, that is, the shear-induced alignment of a-CNFs was preserved over 4 days in HBSS.

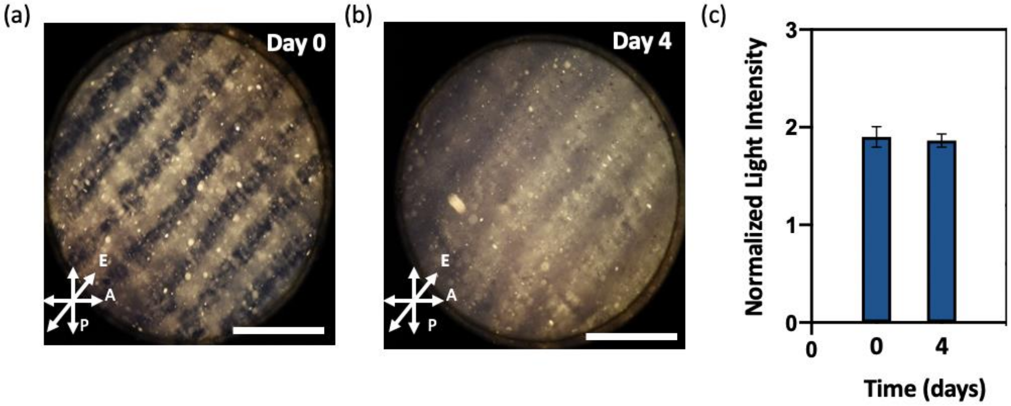

Figure S9. POM images of extruded hydrogels over time. POM images of hydrogel with $C_{a\text{-}CNF}$ = 1.0 wt.% and $C_{Gelatin}$= 4.0 wt.% extruded at Q = 9 mL/min on (a) Day 0 and (b) Day 4 at 37 °C, following incubation in HBSS. The arrows in the image depict the direction of extrusion (E) and the direction of the polarizer (P) and analyzer (A) with respect to the direction of extrusion. (c)

Average normalized light intensities of the maxima peaks in the grey scale profile on Day 0 and Day 4 at 37 °C, following incubation in HBSS. Scale bars are 2 mm.

S10. Normalized Metabolic Activity of Human Dermal Fibroblasts (HDFs) Seeded on or Encapsulated in Hydrogels

The biocompatibility of the a-CNF/gelatin hydrogel was verified for both 2D and 3D culture of HDFs. The metabolic activity of HDFs was measured using PrestoBlue cell viability reagent (Invitrogen). In 2D culture, the HDFs were seeded on the surface of the hydrogels, whereas in 3D culture, the HDFs were encapsulated in the extruded hydrogels. The metabolic activity of HDFs on Day 6 was normalized to that on Day 1. Figure S10a shows the metabolic activity of HDFs seeded on the hydrogels with two different a-CNF concentrations ($C_{a\text{-}CNF}$ of 0.5 or 1.0 wt.% and $C_{gelatin}$ = 4.0 wt.%). In both hydrogels, the HDFs exhibited high normalized metabolic activity, indicating the proliferation of HDFs on the hydrogels. Figure S10b shows the normalized metabolic activity of HDFs encapsulated in the hydrogel with $C_{a\text{-}CNF}$ of 0.5 or 1.0 wt.% and $C_{gelatin}$ = 4.0 wt.%. High viability of over 75% metabolic activity on Day 6 was observed in both cases, indicating good biocompatibility of the hydrogels.

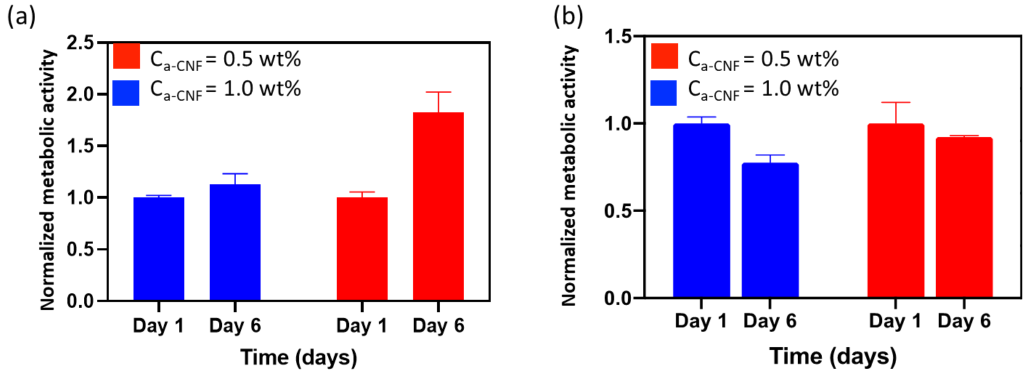

Figure S10. Overtime changes of metabolic activity of HDFs. (a) 2D culture of HDFs on the surface of a-CNF/gelatin hydrogels with different compositions. (b) 3D culture of HDFs encapsulated in a-CNF/gelatin hydrogels with different compositions.

S11. Cell Orientation in the Extruded Gelatin Methacryloyl Hydrogel Threads

Figure S11a shows a representative POM image of the CNF-free gelatin methacryloyl (4 wt.%) hydrogel after 1 min photocrosslinking (λ= 365 nm, intensity = 30 mW cm^{-2}) using 0.1 wt.% lithium phenyl-2,4,6-trimethyl-benzoyl phosphinate (LAP) as a photoinitiator. The thread did not exhibit birefringence, that is, it was not structurally anisotropic. Figure S11b shows a representative fluorescence image of HDFs spreading on the surface of the CNF-free hydrogel. No noticeable cell alignment parallel to the direction of extrusion was observed. The results were also quantified in Figure S11c.

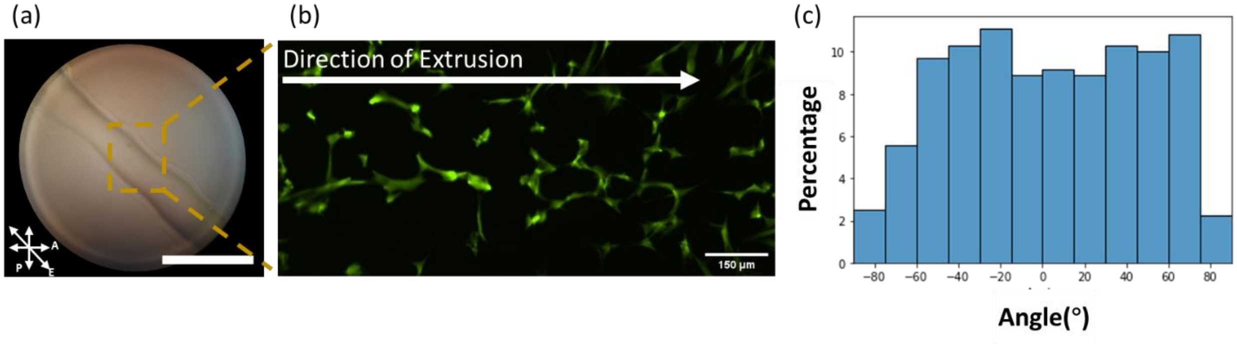

Figure S11. Characterization of HDF orientation on the CNF-free photocrosslinked gelatin methacryloyl hydrogels ($C_{gelatin\ methacryloyl}$ = 4.0 wt.%, C_{LAP} = 0.1 wt.%, 1 min light illumination at 365 nm, 30 mW cm^{-2}). (a) POM image of the extruded hydrogel thread. (b) Fluorescent image of calcium-AM stained HDFs on the hydrogel. (c) Histogram of cell orientation on the hydrogel.

S12. Simulation of Shear Stress Distribution in Different Printheads

Figure S12 shows the results of the simulation of the distribution of the shear stress in the ink during extrusion through two different printheads using COMSOL Multiphysics 5.5 software (COMSOL, Stockholm, Sweden). In the microchannel of the microfluidic printhead (Figure S12a), the shear stress was concentrated in the regions adjacent to the channel walls. The central region of the channel experienced relatively low shear stress. Figure S12b shows a more uniform gradient of shear stress in the nozzle with a circular cross-section, which decreased gradually from the periphery of the nozzle to its center.

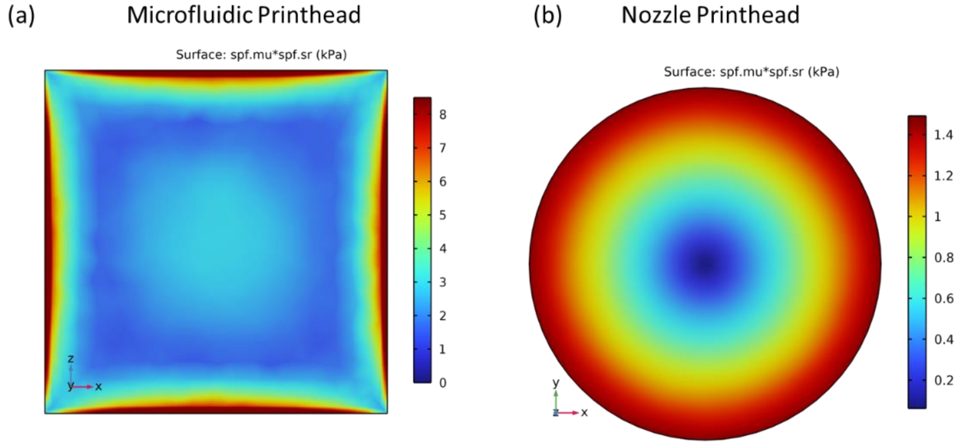

Figure S12. Simulation of the distribution of shear stress in the a-CNF/gelatin ink during extrusion in (a) a channel of the microfluidic printhead and (b) a nozzle printhead. Ink flow rate is Q = 9.0 mL/min for the microfluidic printhead (that is, 0.56 mL/min in each individual channel) and Q = 0.5 mL/min in the nozzle printhead.

References

1. Xue, Yan, Zihao Mou, and Huining Xiao. "Nanocellulose as a Sustainable Biomass Material: Structure, Properties, Present Status and Future Prospects in Biomedical Applications." *Nanoscale* 9, no. 39 (2017): 14758-81.

2. Klemm, Dieter, Friederike Kramer, Sebastian Moritz, Tom Lindström, Mikael Ankerfors, Derek Gray, and Annie Dorris. "Nanocelluloses: A New Family of Nature-Based Materials." *Angewandte Chemie International Edition* 50, no. 24 (2011): 5438-66.
